# Growth Patterns of HIV-Exposed and -Unexposed Infants in African Countries: A Systematic Review and Meta-Analysis

**DOI:** 10.3390/children12050624

**Published:** 2025-05-12

**Authors:** Perpetua Modjadji, Kabelo Mokgalaboni, Wendy N. Phoswa, Tebogo Maria Mothiba, Sogolo L. Lebelo

**Affiliations:** 1 Non-Communicable Diseases Research Unit, South African Medical Research Council, Tygerberg, Cape Town 7505, South Africa; 2 Department of Life and Consumer Sciences, College of Agriculture and Environmental Sciences, University of South Africa, Florida Campus, Johannesburg 1709, South Africa; 3 Faculty of Health Sciences, University of Limpopo, Polokwane 0700, South Africa

**Keywords:** antiretroviral therapy, prevention of mother-to-child transmission, HIV-exposed infants, HIV-unexposed infants, growth patterns, African studies

## Abstract

**Background/Objectives:** The purpose of this study is to understand the prevalence and odds of poor growth patterns among HIV-exposed but uninfected (HEU) versus HIV-unexposed (HUU) infants in the era of antiretroviral therapy (ART) and prevention of mother-to-child transmission (PMTCT) in Africa. **Methods:** We reviewed and meta-analyzed studies on growth patterns among HEU versus HUU infants in Africa. Evidence was gathered from the PubMed and Scopus databases following PRISMA guidelines. We independently evaluated the quality of included studies using Newcastle Ottawa guidelines. Data analysis was performed using an online meta-analysis tool, and the results are reported as odds ratios (OR) and prevalence with 95% confidence intervals (CI). **Results:** A total of 17 studies met the inclusion criteria for this review. The odds of stunting were significantly higher among HEU infants compared to HUU infants, with an odds ratio of 1.56 (95% CI: 1.23–1.97; *p* < 0.01). The pooled prevalence of stunting was 25% (95% CI: 17–33%) in HEU infants and 19% (95% CI: 12–26%) in HUU infants. In contrast, no significant differences were observed for underweight and wasting. The odds of being underweight in HEU infants compared to HUU was 0.85 (95% CI: 0.47–1.56; *p* = 0.60), with a pooled prevalence of 11% (95% CI: 5–17%) in HEU and 14% (95% CI: 5–24%) in HUU. Similarly, the odds of wasting were 1.10 (95% CI: 0.78–1.56; *p* = 0.58), with a pooled prevalence of 9% (95% CI: 3–14%) in HEU and 7% (95% CI: 3–12%) in HUU. **Conclusions:** Stunting was the most prevalent growth deficit among HEU infants compared to their HUU counterparts, with no significant differences observed in the rates of underweight and wasting. To improve postnatal growth outcomes, especially in the evolving landscape of HIV treatment and prevention, efforts should focus on educating and supporting mothers living with HIV.

## 1. Introduction

The global prevalence of people living with HIV (PLWH) was estimated to be approximately (≈) 37.9 million in 2018 [1]. Most of the PLWH reside in Eastern and Southern Africa (≈20.6 million) compared to Central and Western Africa (≈4.5 million) and the Middle East and North Africa (≈180,000) [1,2]. Between 69% and 95% of pregnant women living with HIV (pWLWH) received antiretroviral therapy (ART) to prevent mother-to-child transmission of HIV (PMTCT) and to protect their own health in 2018, compared to 49% reported in 2010 in eastern and southern Africa. The rate of MTCT decreased from 18% in 2010 to 9% in 2018 [1]. The integration of HIV and antenatal care (ANC) services has significantly enhanced the coverage of the three key steps required for PMTCT of HIV, ensuring that over 95% of pregnant women received antenatal testing for HIV and received antiretroviral therapy (ART) [3,4].

This integrated approach is particularly critical in the context of fetal growth, as HIV infection has been associated with impaired placental function, which may contribute to intrauterine growth restriction (IUGR). IUGR is defined as the failure of a fetus to achieve its genetically determined growth potential and is often linked to placental insufficiency, a condition in which the placenta cannot deliver adequate oxygen and nutrients to the fetus [5]. HIV-related placental abnormalities, including inflammation and vascular dysfunction, can compromise placental circulation, thereby increasing the risk of IUGR among HIV-exposed infants [6]. Furthermore, placental insufficiency is a well-established cause of IUGR, and its prevalence is notably higher in pregnancies complicated by maternal infections, including HIV [5]. These findings highlight the importance of early HIV diagnosis and treatment during pregnancy, not only to prevent vertical transmission, but also to support optimal fetal growth and development. Although many pregnant women living with HIV now receive ART, there are still concerns about how this treatment might affect the baby during pregnancy, birth, and after delivery. As a result, more babies are being born HIV-exposed but uninfected (HEU) [7,8,9].

The influence of the maternal intrauterine environment is reflected primarily in the growth parameters (i.e., weight and length) of infants at birth and during the early months of life [10,11,12,13]. However, growth is a dynamic process that continues beyond birth, and adequate infant and childhood nutrition is essential for maintaining healthy growth trajectories. Normal growth patterns reflect overall wellbeing, including nutritional status, health, and socioeconomic conditions [14,15,16]. Conversely, growth failure, often due to undernutrition, is expressed as stunting (low height for age), underweight (low weight for age), and wasting (low weight for length), which indicate insufficient height or weight compared to age-specific standards [17,18,19]. In 2020, an estimated 149 million children under five were stunted, 85 million were underweight, and 45 million were wasted globally [20].

Furthermore, HIV-exposed but uninfected (HEU) children experience a slower phase of linear growth (i.e., stunting) [8], and have an increased risk of morbidity and mortality compared to HIV-unexposed and uninfected (HUU) children [21,22,23]. Particularly among HEU infants, growth outcomes are influenced by a complex interplay of maternal, infant, and environmental factors [24]. Maternal characteristics such as age, health status, and exposure to ART can affect fetal development and postnatal growth [24]. Infant-level factors, including birth weight, sex, and vulnerability to infections, have also been shown to significantly affect growth trajectories in this population [24]. Other factors like where a family lives, how clean their environment is, and what kind of food they have at home also play a significant role in how well these children grow [25].

Studies in Africa examining the postpartum growth of HEU infants have inconsistently reported poorer early growth, high rates of stunting, and underweight among HEU infants [26,27,28], while others reported no differences between HEU infants’ early growth versus HUU [24,29]. Notably, these changes may reflect disruption to the growth hormone axis in infants who are HEU compared with infants who are HUU [30]. From infancy to school age, HEU children experience a slower linear growth [8,31,32], and they have an increased risk of morbidity and mortality compared to their HUU counterparts [21,22,23]. Differences in how babies grow may also come from things like how healthy the mother was during pregnancy, whether she had access to HIV treatment, and whether the family had enough nutritious food [24,33].

Although the literature documents findings on growth patterns or growth failure/faltering (i.e., stunting, underweight and wasting) between HEU and HUU infants, there are inconsistent findings comparing the two groups in African countries. As stated above, HEU infants are susceptible to sub-optimal growth patterns or growth failure, a hallmark of undernutrition expressed as stunting, underweight, and wasting compared to their HUU counterparts. In view of growth failure being a complex phenomenon that carries significant morbidity and mortality implications for infants, we reviewed the existing literature and quantitatively analyzed data to find the overall prevalence and the odds of having growth failure in a comparison between HEU and HUU infants during the era of PMTCT in African countries.

## 2. Methodology

### 2.1. Search Strategy and Literature Search

Due to its nature, this study was conducted and reported in accordance with preferred reporting items for systematic review and meta-analysis (PRISMA) guidelines [34], Supplementary File S1. The protocol for this study was not registered with PROSPERO; however, a thorough search on Cochrane databases was performed to ensure that there was no duplication of the same study. This study identified clinical studies that provided evidence of the prevalence of underweight, stunting, and wasting in Africa. Two independent researchers (KM and WNP) comprehensively searched for evidence on main databases, including PubMed and Scopus, without duration and language restrictions; however, the search was updated on 20 November 2024. The following keywords and MeSH terms were used to source the evidence from the databases: “HIV mother”, “HIV-exposed children”, “HIV-exposed infant”, “child malnutrition”, “infant malnutrition”, “underweight”, “under-nutrition”, “wasting”, “thinness”, and “stunting”. The terms were adapted and applied on PubMed and Scopus using Boolean operators such as “OR” and “AND”. Therefore, the exact searches made on both PubMed and Scopus are presented in Supplementary File S2 Table S1. A manual screening of relevant reviews was also performed to identify additional studies.

### 2.2. Eligibility and Selection Criteria

All retrieved studies were exported to the Mendeley Desktop reference manager (version 1.59.1) for the inclusion and exclusion, which subsequently identified possible duplicates. Before the retrieval of full-text articles, two independent researchers (KM and WNP) thoroughly screened the identified studies using the articles’ titles, abstracts, and keywords. Furthermore, pre-defined eligibility criteria according to the PECOS framework were followed to screen the remaining studies. Briefly, PECOS was as follows: pregnant women, exposure was HIV infection, the comparator was HIV-unexposed uninfected infants, outcomes included underweight, stunting, and wasting, while the study design included cohorts and cross-sectional studies. Any disagreements that arose were resolved with the input of a third independent researcher (PM) who made a final decision on the selection of studies in question. Therefore, all studies that reported on underweight, wasting, and stunting published in English from the database’s inception to 20 November 2024 were included. All studies on HIV-exposed children born in Africa were included. The studies included were cross-sectional and cohort. The prevalence of stunting, underweight, and wasting was defined as height for age, weight for age, and weight for height Z-scores, respectively. However, studies conducted primarily on children with other medical conditions, chronic diseases, randomized controlled trials, and studies not reporting data as numbers and prevalence were not considered. When the same study was published by the same author, we considered the initial cohorts to avoid data duplication.

### 2.3. Data Extraction

KM developed an Excel data extraction sheet, which included the author, year of publication, country, study design, sample size, and the proportion of underweight, stunting, and wasting infants. Two researchers (KM and PM) independently extracted data from all relevant studies in accordance with their extraction sheet. The two sheets were shared with a third independent researcher (WNP) to assess the accuracy and consistency of the extracted data, and any notable disagreements were resolved through discussion with primary researchers (KM and PM), who extracted the data to reach a final decision.

### 2.4. Quality Assessment

Two researchers (WNP and KM) reviewed the quality of each study on their own using the Newcastle-Ottawa checklist [35]. They rated the studies as high, moderate, or low quality based on how many stars they received: 7 or more stars meant high quality, 4 to 6 stars meant moderate quality, and fewer than 4 stars meant low quality. If they did not agree on a rating, a third researcher (PM) helped make the final decision.

### 2.5. Data Analysis

The extracted data were analyzed using an online meta-analysis software: https://metaanalysisonline.com/ (accessed on 11 December 2024). Random and fixed-effect models were used based on the level of heterogeneity. The number of infants in the HIV-exposed uninfected and HIV-unexposed groups that had developed growth patterns (underweight, stunting, and wasting) and the total sample sizes in both groups were used to estimate the overall effect estimates. Data are reported as the odds ratio (OR) and 95% confidence intervals. The prevalence of these growth patterns was also determined. A probability of less than 5% was considered statistically significant. Statistical heterogeneity was assessed using the *I*^2^ and Chi-square statistics [36,37]. The *I*^2^ statistic values of 0, 25, 50, and 75% indicate zero, low, moderate, and high heterogeneity, respectively. Based on the region of publication in Africa, subgroup analysis was performed to find the source of the observed heterogeneity in case *I*^2^ was above 50%. Furthermore, if more than ten studies were available, a funnel plot and Egger’s regression tests were used to assess publication bias among the included studies [38].

## 3. Results

### 3.1. Databases and Literature Search

**Figure 1:**
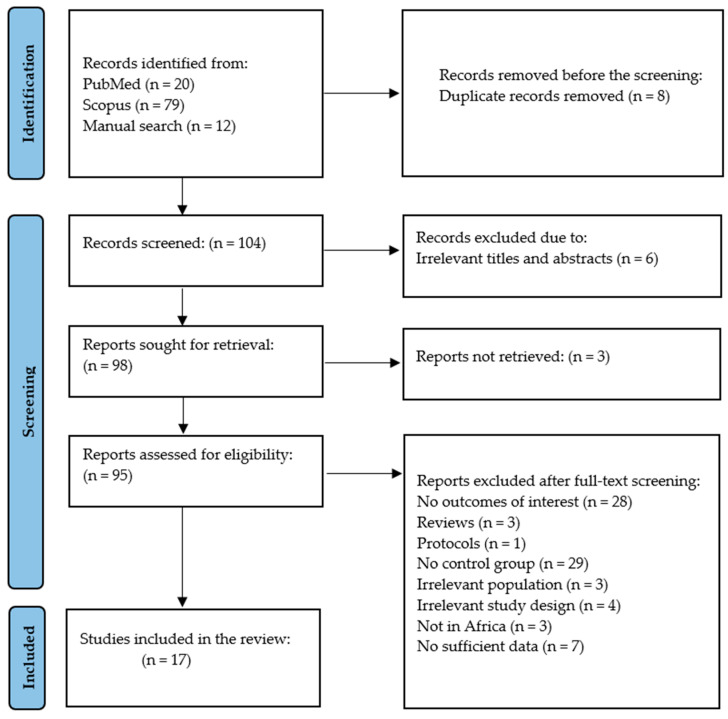
A Preferred Reporting Items for Systematic Review and Meta-Analysis (PRISMA) flow chart representing the selection of studies.

The evidence was retrieved from two databases, with 21 records from PubMed, 79 from Scopus, and twelve retrieved via manual screening of relevant studies. Therefore, 112 records were retrieved and critically assessed for inclusion. Briefly, eight were identified by the reference manager as duplicates and were thus excluded. An additional six were excluded at the early screening stage due to irrelevant titles, abstracts, keywords, and the overall focus. In total, 3 of the 104 remaining records were unavailable; however, attempts were made to obtain these full texts by contacting the primary investigators, which proved fruitless. Amongst the 95 that were retrieved, 78 articles were excluded based on the following reasons: (1) irrelevant population, (2) single-armed study without a control group, (3) studies not conducted in Africa (2 from China and 1 from India), (4) review articles, (5) randomized controlled trials, (6) data not reported as prevalence, (7) protocols, and (8) no outcome of interest. Hence, only 17 [28,39,40,41,42,43,44,45,46,47,48,49,50,51,52,53,54] were included in the meta-analysis based on the available data (Figure 1).

### 3.2. Quality of Studies

The studies included in the review were generally of good quality, based on the Newcastle-Ottawa Scale. Most cohort studies scored well, with eight of them earning 8 stars and one scoring 6 (see Supplementary File S2, Table S2). All the cross-sectional studies also performed well, each receiving 7 stars (see Supplementary File S2, Table S3).

### 3.3. General Characteristics of the Included Studies

All included studies [28,39,40,41,42,43,44,45,46,47,48,49,50,51,52,53,54] were published in peer-reviewed journals between 2012 and 2023. All studies were conducted in different parts of Africa, ranging from the Southern, Eastern, and Western regions. Briefly, these included South Africa [28,40,41,48,50,52], Botswana [42], Uganda [39], Kenya [46,47], Zimbabwe [44,45], Ethiopia [49,53], Nigeria [43], and Malawi [51,54]. We included cohorts (prospective, retrospective, and longitudinal observational) as well as cross-sectional studies. A detailed overview is presented in Table 1.

### 3.4. Meta-Analysis Findings

#### 3.4.1. Underweight in HIV-Exposed Infants Compared to Unexposed-Uninfected Infants

**Figure 2:**
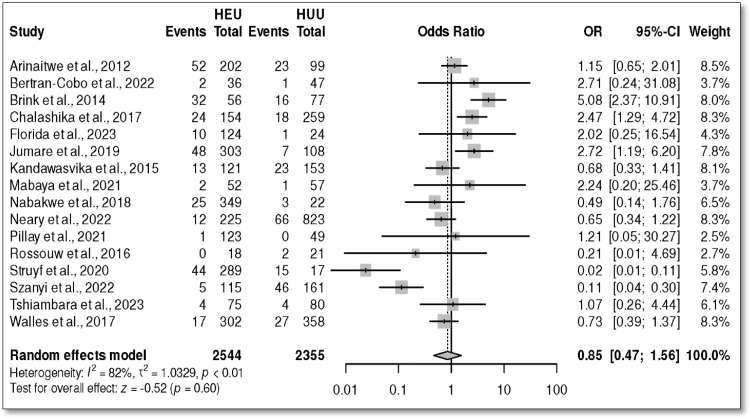
Odds ratio in weight for age Z-score (WAZ)/underweight in HIV-exposed uninfected versus HIV-unexposed infants in Africa [28,39,40,41,42,43,44,45,46,47,48,49,51,52,53,54].

**Figure 3:**
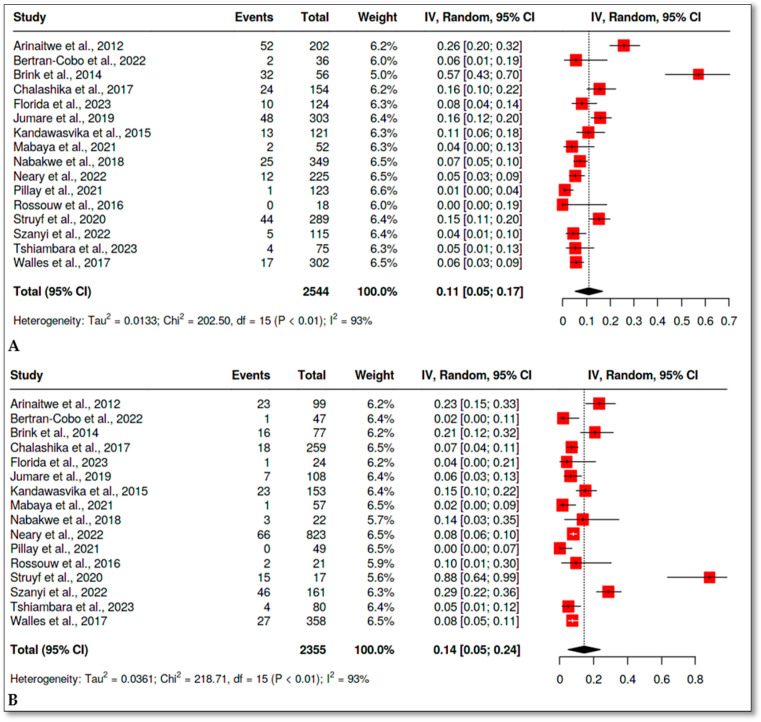
Prevalence of underweight in HEU and HUU infants. (**A**) Random effect meta-analysis showing the prevalence of underweight in HEU infants. (**B**) Random effect meta-analysis showing the prevalence of underweight in HUU infants [28,39,40,41,42,43,44,45,46,47,48,49,51,52,53,54].

A total of 16 studies [28,39,40,41,42,43,44,45,46,47,48,49,51,52,53,54] reported underweight in both groups. Only, one study [50] reported no events in either group; thus, it was not included in the meta-analysis. The overall OR was 0.85 with a 95% CI (0.47, 1.56), *p* = 0.60 (Figure 2). However, the studies revealed high heterogeneity (*I*^2^ = 82%). The results for prevalence are presented in Figure 3. The prevalence in the HEU group was 11%, 95% CI (5, 17) (Figure 3A), and it was 14%, 95% CI (5, 24) in the HUU group (Figure 3B).

#### 3.4.2. Stunting in HIV-Exposed Uninfected Infants Compared to Unexposed Infants

**Figure 4:**
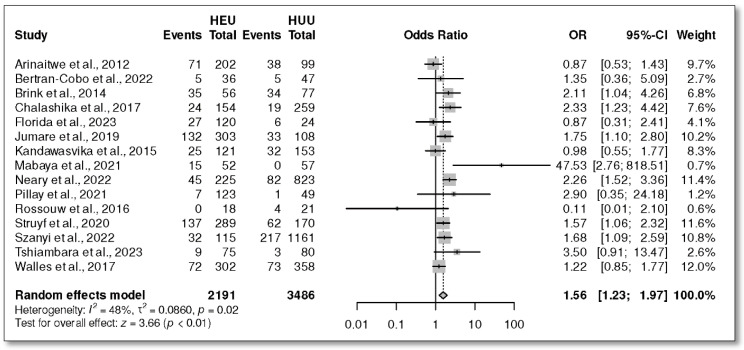
Odds ratio of length for age Z-score (LAZ)/stunting in HEU versus HUU infants [28,39,40,41,42,43,44,45,47,48,49,51,52,53,54].

**Figure 5:**
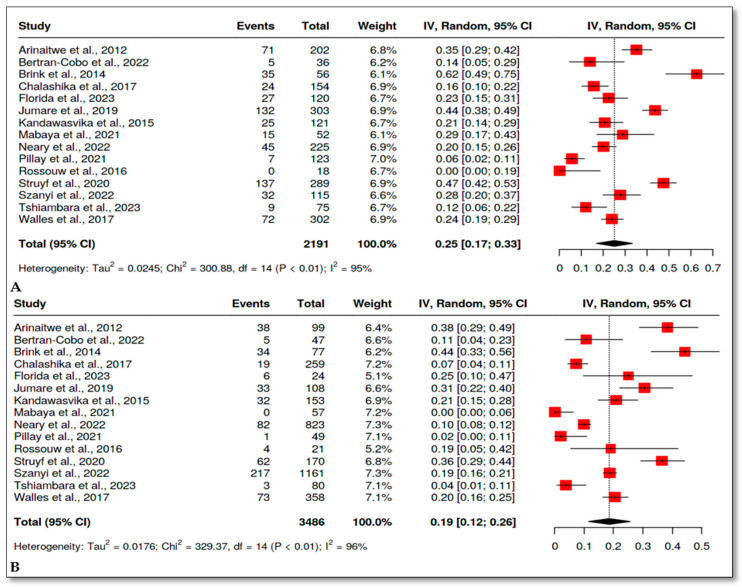
Prevalence of stunting in HEU and HUU. (**A**) Random effect meta-analysis showing the prevalence of stunting in HEU infants. (**B**) Random effect meta-analysis showing the prevalence of stunting in HUU infants [28,39,40,41,42,43,44,45,47,48,49,51,52,53,54].

Fifteen studies [28,39,40,41,42,43,44,45,47,48,49,51,52,53,54] were included in the meta-analysis of stunting in HIV-exposed uninfected and HIV-unexposed infants. The overall OR was 1.56 with a 95% CI (1.23, 1.97), *p* ˂ 0.01 (Figure 4). The prevalence in the HEU group was 25%, 95% CI (17, 33) (Figure 5A), and it was 19%, 95% CI (12, 26) in the HUU group (Figure 5B).

#### 3.4.3. Wasting in HIV-Exposed Infants Compared to Unexposed-Uninfected Infants

**Figure 6:**
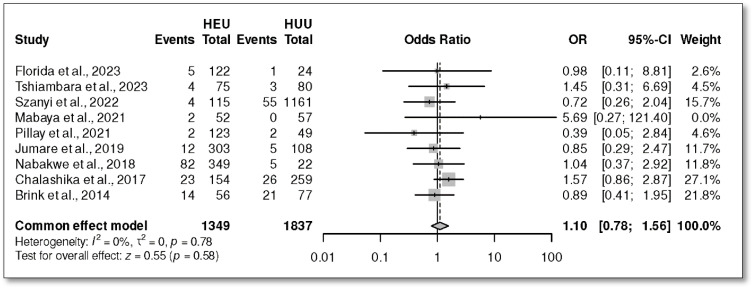
The odds ratio on weight for length Z-score (WLZ)/wasting in HEU versus HUU infants in Africa [28,41,42,43,45,46,49,52,54].

**Figure 7:**
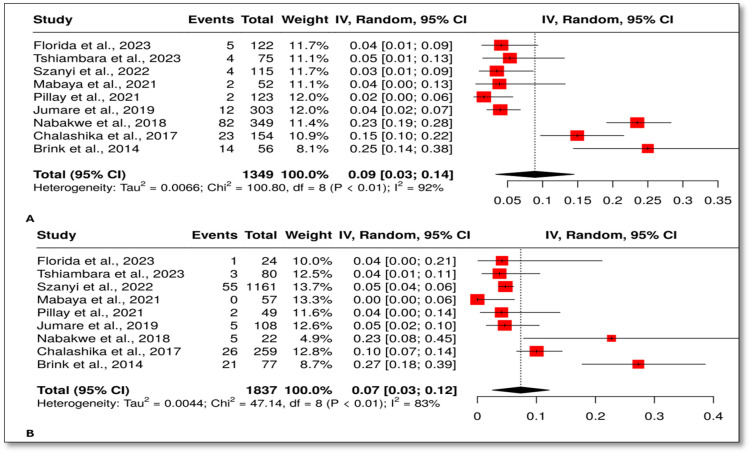
Prevalence of wasting in HEU and HUU. (**A**) Random effect meta-analysis showing the prevalence of wasting in HEU infants. (**B**) Random effect meta-analysis showing the prevalence of wasting in HUU infants [28,41,42,43,45,46,49,52,54].

A total of nine studies were included in the meta-analysis of wasting in HEU and HUU infants. The overall OR was 1.10 with a 95% CI (0.78, 1.56), *p* = 0.58 (Figure 6). Significant heterogeneity was not observed (*I*^2^ = 0%, *p* = 0.78), suggesting that the effect sizes across studies were uniform in both magnitude and direction. The prevalence of wasting in those exposed was 9%, 95% CI (3, 14) (Figure 7A), and it was 7%, 95% CI (3, 12) (Figure 7B).

#### 3.4.4. Graphical and Statistical Assessment of Publication Bias

For studies that assessed underweight, the funnel plot did not indicate a potential publication bias (Supplementary File S2, Figure S1A). The Egger’s test does not support the presence of funnel plot asymmetry. For those that assessed stunting, the funnel plot did not indicate a potential publication bias (Supplementary File S2, Figure S1B). The Egger’s test does not support the presence of funnel plot asymmetry. Similarly, for studies that assessed wasting, the funnel plot showed no potential publication bias (Supplementary File S2, Figure S1C). The Egger’s test also does not support the presence of funnel plot asymmetry.

## 4. Discussion

This study synthesizes the published evidence to evaluate the prevalence and odds of developing growth failure among HEU and HUU infants in the era of PMTCT. The findings show a significant concern: HEU infants are at a notably higher risk of stunting compared to their HUU counterparts. Recent evidence shows that HEU infants have 56% higher odds of being stunted, reflecting chronic undernutrition and long-term growth failure [55,56,57,58]. Stunting, defined as low height for age, is a cumulative indicator of poor nutrition and repeated infections, particularly during the first 1000 days of life [57,58,59]. Unlike wasting or underweight, which may reflect acute nutritional deficits, stunting is often the result of prolonged exposure to adverse conditions, including poor maternal health and inadequate infant feeding practices [56,58]. The elevated risk of stunting in HEU infants highlights the need for targeted nutritional and health interventions to mitigate these adverse outcomes.

Furthermore, this elevated risk among HEU infants may be attributed to a complex interplay of biological and environmental factors. In utero exposure to HIV ART, even in the absence of infection, may disrupt fetal growth and immune development [56,57,58,60]. Studies suggest that HIV-exposed fetuses may experience altered placental function, inflammation, or oxidative stress, which can impair nutrient transfer and fetal growth [55,58,60,61]. These biological disruptions are compounded by maternal health challenges, including ART side effects and immune dysregulation, which may further compromise fetal development.

Postnatally, the health and nutritional status of the mother continues to play a pivotal role. Mothers living with HIV often face food insecurity, limited access to healthcare, and increased susceptibility to infections, all of which can affect breastfeeding practices and caregiving environments [56,57,58,61]. Food insecurity has been shown to significantly impact maternal dietary diversity and breastfeeding outcomes, especially in low-resource settings [13,62,63,64]. These challenges are often exacerbated by broader socioeconomic disadvantages, including poverty, inadequate sanitation, and limited access to clean water and health services, all of which are known contributors to stunting [59,60,62,63]. Despite WHO recommendations supporting breastfeeding for mothers living with HIV on ART, implementation remains inconsistent in many settings due to systemic barriers and stigma [65,66,67].

Importantly, the lack of significant differences in underweight and wasting between HEU and HUU infants in recent studies suggests that the growth deficits in HEU infants are more chronic than acute [55,56,60]. This pattern emphasizes the need for early and sustained interventions that go beyond HIV prevention and treatment [57,58,59,61]. Nutritional support, maternal health services, and social protection programs tailored to the needs of HEU infants, and their families are essential to address the root causes of stunting [56,57,58,59].

This study has several strengths, including a comprehensive search strategy conducted independently across major databases and the inclusion of studies from diverse African regions. However, there are important limitations to consider when interpreting the findings. Although Africa bears a significant burden of global HIV prevalence, only 17 relevant studies met the inclusion criteria, limiting the generalizability of the results. While subgroup analyses were performed to explore regional variations, heterogeneity in outcomes remained. A key limitation is that most included studies did not specify whether the HIV in pregnant mothers was well controlled or uncontrolled, nor did they consistently report maternal viral load or CD4 count. This lack of stratification may have masked important differences in infant growth outcomes between these subgroups. Additionally, the form and timing of ART used during pregnancy were often not reported, making it difficult to assess the influence of specific ART regimens on fetal and postnatal growth. Furthermore, many studies did not account for other critical factors known to influence fetal growth, such as maternal nutritional status, co-infections, socioeconomic conditions, and environmental exposures. The absence of these variables in the analysis may have introduced residual confounding. Despite the overall good quality of studies based on the Newcastle-Ottawa Scale, these methodological gaps highlight the need for more detailed and standardized reporting in future research.

## 5. Conclusions and Recommendations

Despite the success of PMTCT programs in reducing vertical HIV transmission, our systematic review and meta-analysis shows that HEU infants remain significantly more vulnerable to stunting than their HUU counterparts, highlighting a critical gap in child health strategies. This persistent growth deficit emphasizes the need for a holistic response that includes targeted nutritional interventions, routine health monitoring, such as the consistent use of the Road to Health Book, and socioeconomic support to address the underlying determinants of undernutrition. Although the WHO recommends breastfeeding for mothers on ART, implementation is often hindered by stigma, misinformation, and systemic barriers, particularly in resource-limited settings. To ensure that HEU infants thrive, future policies must integrate these insights and prioritize longitudinal research to better understand and address the complex drivers of stunting in this vulnerable population. In addition, efforts should focus on educating and supporting mothers living with HIV to improve postnatal growth outcomes for their infants, especially within the evolving landscape of HIV treatment and prevention.

## Figures and Tables

**Table 1:** The characteristics of the included studies on growth patterns between HIV-exposed and -unexposed uninfected infants.

Author	Country	Study Design	Sample Size and Participant Characteristics	Outcomes	Prevalence in HEU (%)	Prevalence in HUU n (%)
Arinaitwe et al., 2012 [39]	Uganda	Prospective, longitudinal cohort	202 HIV-exposed uninfected (HEU) followed for 2 years. 99 HIV-unexposed uninfected (HUU)	Underweight	52 (26)	23 (23)
				Stunting	71 (35)	38 (38)
				Wasting	NR	NR
Bertran-Cobo et al., 2022 [40]	South Africa	Complete case–cohort	36 HEU followed for 2 years. 47 HUU	Underweight	2 (6.45)	1 (2.44)
				Stunting	5 (16.13)	5 (12.19)
				Wasting	0 (0)	0 (0)
Brink et al., 2014 [41]	South Africa	Cross-sectional	56 HEU followed for 18 months. 77 HUU	Underweight	32 (57.1)	16 (20.8)
				Stunting	35 (62.5)	34 (44.2)
				Wasting	14 (25)	21 (27.3)
Chalashika et al., 2017 [42]	Botswana	Cross-sectional	154 HEU followed for 24 months. 259 HUU	Underweight	24 (15.6)	18 (6.9)
				Stunting	24 (15.6)	19 (7.3)
				Wasting	23 (14.9)	26 (10)
Floridia et al., 2023 [54]	Malawi	Prospective cohort	HEU 124, 120, 122 after 12 months of follow-up 24 HUU followed for 12 months	Underweight	10 (8.1)	1 (4.2)
				Stunting	27 (22.5)	6 (25)
				Wasting	5 (4.1)	1 (4.2)
Jumare et al., 2019 [43]	Nigeria	Prospective cohort	303 HEU followed for 188 months. 108 HUU	Underweight	48 (15.8)	7 (6.2)
				Stunting	132 (44.3)	33 (30.9)
				Wasting	12 (4.9)	5 (4.9)
Kandawasvika et al., 2015 [44]	Zimbabwe	Cross-sectional	121 HEU 153 HUU	Underweight	13 (11)	23 (15)
				Stunting	25 (21)	32 (21)
				Wasting	NR	NR
Mabaya et al., 2021 [45]	Zimbabwe	Prospective cohort	52 HEU followed for 16 weeks. 57 HUU	Underweight	2 (3.85)	1 (1.75)
				Stunting	15 (28.85)	0 (0)
				Wasting	2 (3.85)	0 (0)
Nabakwe et al., 2018 [46]	Kenya	Cross Sectional	349 HEU 22 HUU	Underweight	25 (89.3)	3 (10.7)
				Stunting	NR	NR
				Wasting	82 (94.3)	5 (5.7)
Neary et al., 2022 [47]	Kenya	Cross-sectional	225 HEU with a 9-month follow-up 823 HUU	Underweight	12 (27)	66 (8)
				Stunting	45 (20)	82 (10)
				Wasting	NR	NR
Pillay et al., 2021 [28]	South Africa	Cohort	123 HEU with a 9-month follow-up 49 HUU	Underweight	1 (0.82)	0 (0)
				Stunting	7 (5.74)	1 (20)
				Wasting	2 (1.64)	2 (40)
Rossouw et al., 2016 [48]	South Africa	Prospective cohort	18 HEU with an 18-month follow-up 21 HUU	Underweight	0 (0)	2 (10)
				Stunting	0 (0)	4 (19)
				Wasting	0 (0)	0 (0)
Szanyi et al., 2022 [49]	Ethiopia	Prospective Cohort	115 HEU with 18 months follow-up 1161 HUU	Underweight	5 (4.35)	46 (3.96)
				Stunting	32 (27.83)	217 (18.69)
				Wasting	4 (3.48)	55 (4.74)
Springer et al., 2020 [50]	South Africa	Prospective Cohort	32 HEU 27 HUU	Underweight	0 (0)	0 (0)
				Stunting	2 (6)	4 (14.8)
				Wasting	NR	NR
Struyf et al., 2020 [51]	Malawi	Prospective Cohort	289 HEU 170 HUU	Underweight	44 (15.2)	15 (8.5)
				Stunting	137 (46)	62 (36.5)
				Wasting	NR	NR
Tshiambara et al., 2023 [52]	South Africa	Cross-sectional	75 HEU with 12 months follow-up 80 HUU	Underweight	4 (5.5)	4 (5.1)
				Stunting	9 (12.3)	3 (3.8)
				Wasting	4 (5.5)	3 (3.9)
Walles et al., 2017 [53]	Ethiopia	Cross-sectional	302 HEU 358 HUU	Underweight	17 (5.7)	27 (6.7)
				Stunting	72 (25.1)	73 (20.5)
				Wasting	NR	NR

HEU: HIV-exposed uninfected, HUU: HIV-unexposed uninfected, NR: not reported.

## Data Availability

The original contributions presented in the study are included in the article/Supplementary Materials, further inquiries can be directed to the corresponding author.

## Supplementary Materials

The following supporting information can be downloaded at: https://www.mdpi.com/article/10.3390/children12050624/s1, Supplementary File S1: PRISMA checklist; Supplementary File S2 Table S1: Search strategy; Supplementary File S2 Table S2: Quality assessment for cohorts; Supplementary File S2 Table S3: Quality assessment for cross-sectional studies; Figure S1: Funnel plot showing assessment of publication bias. A: underweight, B: stunting, C: wasting.
