# Supplementary material for: Growth Patterns of HIV-Exposed and -Unexposed Infants in African Countries: A Systematic Review and Meta-Analysis
Source: Children (Basel). 2025 May 12;12(5):624. doi: 10.3390/children12050624 (PMC12110401; doi:10.3390/children12050624)
Supplement: Supplementary file 1 [file children-12-00624-s001.zip › Supplementary File S2.pdf]

**Supplementary File S2**

**Table S1:** Search strategy adapted on databases.

| Database | Search | Records |
|---|---|---|
| **PubMed** | ((HIV mothers[MeSH Terms]) AND ((HIV exposed children[MeSH Terms]) OR (HIV exposed infant[MeSH Terms]))) AND ((((((child malnutrition[MeSH Terms]) OR (infant malnutrition[MeSH Terms])) OR (Undernutrition[MeSH Terms])) OR (Stunting[MeSH Terms])) OR (Wasting[MeSH Terms])) OR (thinness[MeSH Terms])) | 20 |
| **Scopus** | ( TITLE-ABS-KEY ( hiv AND mother ) AND TITLE-ABS-KEY ( hiv-exposed AND children ) OR TITLE-ABS-KEY ( hiv-exposed AND infant ) AND TITLE-ABS-KEY ( child AND malnutrition ) OR TITLE-ABS-KEY ( infant AND malnutrition ) OR TITLE-ABS-KEY ( undernutrition ) OR TITLE-ABS-KEY ( stunting ) OR TITLE-ABS-KEY ( thinness ) OR TITLE-ABS-KEY ( wasting ) ) AND ( LIMIT-TO ( DOCTYPE , "ar" ) ) | 79 |

**Table S2: Quality assessment of Cohort Studies**

| | Selection | | | | Comparibility | Outcomes | | | Score |
|---|---|---|---|---|---|---|---|---|---|
| Authors | Representativeness of the exposed cohort | Selection of thenon-exposed cohort | Ascertainmentof exposure | Demonstration that outcome of interest was not present at start of study | Comparability ofcohorts on the basis of the design or analysis | Assessmentof outcome | Was follow-up long enough for outcomes to occur | Adequacy of follow up of cohorts | |
| Arinaitwe et al 2012 | ★ | ★ | ★ | ★ | ★ | ★ | ☆ | ☆ | 6 |
| Bertran et al 2022 | ★ | ★ | ★ | ★ | ★ | ★ | ★ | ★ | 8 |
| Jumare et al 2019 | ★ | ★ | ★ | ★ | ★ | ★ | ★ | ★ | 8 |
| Mabaya et al 2021 | ★ | ★ | ★ | ★ | ★ | ★ | ★ | ★ | 8 |
| Pillay et al 2021 | ★ | ★ | ★ | ★ | ★ | ★ | ★ | ★ | 8 |
| Rossouw et al 2016 | ★ | ★ | ★ | ★ | ★ | ★ | ★ | ★ | 8 |
| Springer et al 2020 | ★ | ★ | ★ | ★ | ★ | ★ | ★ | ★ | 8 |
| Struyf et al 2020 | ★ | ★ | ★ | ★ | ★ | ★ | ★ | ★ | 8 |
| Szanyi et al 2022 | ★ | ★ | ★ | ★ | ★ | ★ | ★ | ★ | 8 |
| Florida et al., 2023 | ★ | ★ | ★ | ★ | ★ | ★ | ★ | ★ | 8 |

**Table S3**: Quality of included Cross-sectional Studies

| Authors | Selection | | | | Comparability | Outcome | | Score |
|---|---|---|---|---|---|---|---|---|
| | Representativeness of the sample | Sample size | Nonrespondents | Ascertainment of exposure | Based on design and analysis | Assessment of outcome | Statistical test | |
| Brink et al 2014 | ★ | ★ | ☆ | ★ | ★★ | ★ | ★ | 7 |
| Chalashika et al 2017 | ★ | ★ | ☆ | ★ | ★★ | ★ | ★ | 7 |
| Kandawasvika et al 2015 | ★ | ★ | ☆ | ★ | ★★ | ★ | ★ | 7 |
| Neary et al 2022 | ★ | ★ | ☆ | ★ | ★★ | ★ | ★ | 7 |
| Nabakwe et al., 2018 | ★ | ★ | ☆ | ★ | ★★ | ★ | ★ | 7 |
| Tshiambara et al., 2023 | ★ | ★ | ★ | ★ | ★★ | ★ | ★ | 8 |
| Walles et al 2017 | ★ | ★ | ☆ | ★ | ★★ | ★ | ★ | 7 |

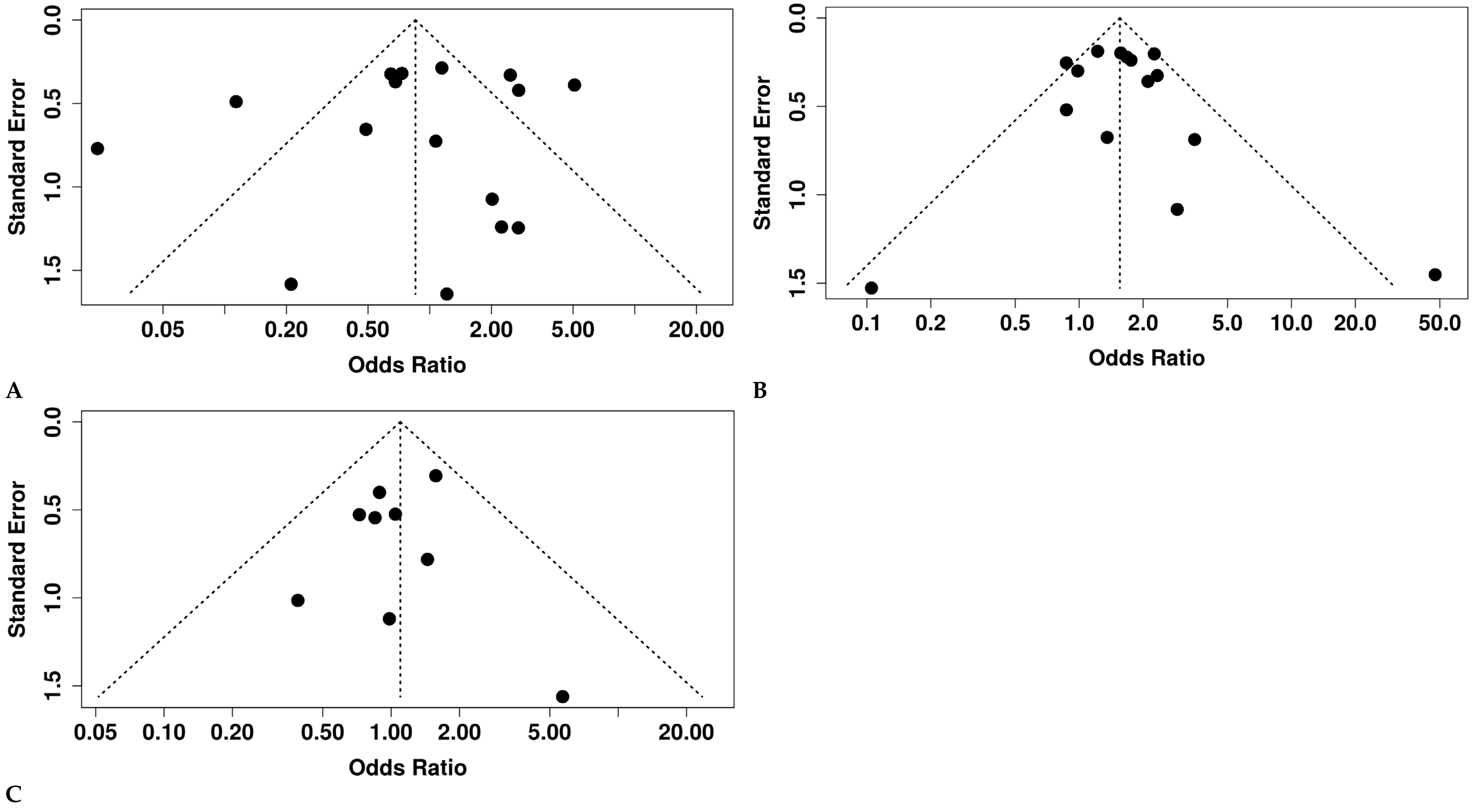


**Figure S1:** Funnel plot showing assessment of publication bias. A: underweight, B: stunting, C: wasting.
